# QPL-enabled HTSlib library: accelerating sequence file compression using Intel IAA

**DOI:** 10.1093/bioadv/vbag250

**Published:** 2026-08-26

**Authors:** Benjamin Laflen, Zheyu Li, Maxwell Urbina, Tajana Rosing, Niema Moshiri

**Affiliations:** Department of Computer Science & Engineering, UC San Diego, La Jolla, CA 92093, United States; Department of Computer Science & Engineering, UC San Diego, La Jolla, CA 92093, United States; Department of Computer Science & Engineering, UC San Diego, La Jolla, CA 92093, United States; Department of Computer Science & Engineering, UC San Diego, La Jolla, CA 92093, United States; Department of Computer Science & Engineering, UC San Diego, La Jolla, CA 92093, United States

## Abstract

**Summary:**

Sequence analysis workflows require the accessibility of large datasets, which require state-of-the-art compression tools. These compression tools, such as Samtools, often rely on HTSlib as a GZip implementation, but are still limited by throughput on time-intensive compression. QPL-HTSLib offers order of magnitude speedups for the compression and decompression of the SAM and BAM file formats commonly used in genomics workflows at the cost of a slightly larger compressed file, and is a drop-in replacement for HTSlib on Intel systems.

**Availability and implementation:**

QPL-HTSLib is freely available on Github as an open-source software project.

## 1 Introduction

Nearly every modern bioinformatics workflow starts with generating read data in wet labs and mapping those reads onto a reference genome to enable the detection of small mutations and anomalies in the subject population (Canzar and Salzberg 2017). This read mapping alignment generates a SAM (Sequence Alignment/Map format) file containing the sequence and difference data (Li *et al.* 2009). Due to the amount of sequence data generated by modern experiments, however, these SAM files, which are stored using plaintext base encodings, can easily reach into the terabytes in size (the 1000 Genomes Project, one large-scale sequencing project, had compiled over 200TB of total data by 2012 (Waltz 2012)). Thus for ease of transport and manipulation, SAM files must be compressed into much smaller lossless BAM (Binary Alignment/Map format) files (Li *et al.* 2009). The lossy CRAM format has also become popular for transporting the same data (Cochrane *et al.* 2013).

As the canonical C library for accomplishing both the read mapping and this compression, Samtools is vital for any modern workflow that involves gene sequencing (Li *et al.* 2009). HTSlib was developed as an upgraded architecture for Samtools in order to accelerate and more readily enable many common bioinformatics workflows. It is structured as a cascade of successive layers, with the primary compression happening in the BGZF (Blocked GNU Zip Format) block compression layer, built on Deflate compression. HTSLib itself utilizes one of two underlying system libraries for its Deflate compression, prioritizing the faster Libdeflate (Biggers 2020) library where available, and otherwise defaulting to Zlib (Deutsch and Gailly 1996), as seen in the original paper (Bonfield *et al.* 2021). Using Libdeflate has been shown to increase the compression rate of HTSLib by up to 2.3 × (Siebelt 2024). Additionally, as zlib is linked at compile time, different API-compatible libraries such as zlib-ng (a modern fork of zlib optimized for modern CPUs) (Rosbach n.d.) can be used as drop-in replacements, although they must conform to the exact zlib API. However, given the sizes of the files involved, compressing massive datasets remains a barrier to quickly using sequence data.

Intel’s IAA (In-Memory Analytics Accelerator) is a purpose-built accelerator designed to offload various database tasks from the CPU to reduce processor load while greatly speeding them up (Intel Corporation 2023). Intel’s QPL (Query Processing Library) is the open-source library of choice for carrying out high-performance operations on Intel CPUs, and is C-compatible. The libary is designed to optimize database operations, such as Deflate compression/decompression, on all Intel chips, while offering even greater efficiency via automatic usage of IAA where present. These are reflected in its primary two operating modes: the “software path” utilizes strictly in-software optimizations, while the “hardware path” also utilizes on-chip IAA devices if present (Intel Corporation 2021).

Here we introduce QPL-HTSLib, a combination of HTSLib and QPL which greatly accelerates SAM/BAM compression and decompression, along with smaller acceleration for CRAM, using IAA and highly optimized QPL processes. Using these optimized algorithms, QPL-HTSLib offers order of magnitude speedup on Intel chips over existing standard HTSLib builds and can serve as a drop-in replacement where applicable for Samtools and Bioinformatics workflows at large.

## 2 Results and discussion

QPL-HTSLib is a library written in C++ that functions as an alternative implementation of HTSlib. Like HTSLib, QPL-HTSLib depends on zlib. While QPL-HTSLib also depends on QPL usability and is thus supported only on the Intel 64 Platform, IAA availability is not necessary (Intel Corporation 2021) (a compiled copy of QPL is included in the QPL-HTSLib repository for convenience). While QPL-HTSLib supports all of the same use-cases and applications as HTSlib generally, QPL usage was added specifically to the BGZF compression and decompression algorithms, and so QPL-HTSLib is intended for workflows utilizing HTSlib for those purposes, such as SAM-to-BAM compression with Samtools. Other compression algorithms such as LZMA (Lempel–Ziv–Markov chain algorithm) that do not use Deflate lie outside the scope of QPL optimization and are not built into IAA acceleration (Intel Corporation 2023). While the benefits of QPL-HTSLib are most readily apparent with highly deflate-based SAM/BAM decompression and compression, QPL-HTSLib also has an optimized CRAM format implementation. Internally, the CRAM specification utilizes some Deflate logic, and that segment is also offloaded to QPL, although it is a smaller portion than with the SAM/BAM format.

QPL-HTSLib attaches to the existing HTSlib process by intercepting the low-level Deflate streams that will be used for compression or decompression and redirecting them to QPL rather than zlib or libdeflate. QPL handles the low-level Deflate implementation, while the HTSlib wrapping is preserved for interoperability. Files compressed with QPL-HTSLib are fully standard-compliant and can be decompressed by any process supporting standard HTSlib. Due to IAA’s limited history buffer size, however, QPL is only capable of decompressing files with a history buffer no larger than 4 kb (Intel Corporation 2021). In practice, this means QPL-HTSLib will only accelerate the decompression of files compressed by the library; in other decompression cases, QPL-HTSLib falls back to zlib HTSlib logic. This is also one of the two primary reasons QPL-HTSLib was implemented as a fork of HTSLib and QPL wasn’t simply wrapped in a shim like zlib-accel (a zlib API-compatible shim for QPL) (Intel Corporation 2025). Because QPL doesn’t quite conform to the Deflate and Zlib API standards, linking one of these shims against HTSLib would cause an error in any case where the file was compressed with a standard history buffer. Forking HTSLib allowed us to gracefully fall back to any other drop-in linked zlib library of the user’s choice using the existing linking mechanism instead of just erroring immediately, increasing the safety and security of the application. Our other motivation for attaching within HTSLib as opposed to linking is that we were able to tune the whole call site to maximize compatibility with QPL. Stacking shims upon APIs promises to add unnecessary overhead and conversions, which is undesirable for such a hot path and which QPL-HTSLib cleanly side-steps. In turn, QPL-HTSLib exposes an identical API to Standard HTSLib. Thus, Samtools or any other application that uses HTSLib can be easily linked at compile time to QPL-HTSLib (or back) by simply passing the compiled QPL-HTSLib library as the relevant argument.

To validate QPL and IAA as the best underlying Deflate solution to use for optimizing HTSLib, we compared QPL’s raw compression and decompression speed in both hardware and software mode against several state of the art CPU-based Deflate implementations configured into their fastest but least efficient modes. Libdeflate used LIBDEFLATE_LEVEL = 1, the fastest compressing mode (level 0 yields no compression) (Biggers 2020). Zlib and zlib-ng used Z_BEST_SPEED (Deutsch and Gailly 1996). ISA-L (Intel’s Intelligent Storage Acceleration Library) was configured to use ISAL_LEVEL = 0, which unlike level 0 for libdeflate and zlib is a compressive level (Intel Corporation 2017). The six libraries were tested for compression and decompression speed, and compression ratio, against a set of actual genomic data. We used the NA12878 Oxford Nanopore Technologies (ONT) sequence dataset from the Illumina Platinum Genomes (Eberle *et al.* 2017) and mapped to the GRCh38 human reference genome using Minimap2 v2.24-r1122 (Li 2018), obtained from 42basepairs (42basepairs n.d.). This is a 750GB BAM file representative of what HTSLib is typically run against. As can be seen in Table 1, QPL in its software path is on par with the best Deflate libraries, significantly beating libdeflate on compression speed but losing on decompression speed and compression ratio, while slightly behind ISA-L in all three major benchmarks. This makes sense, as ISA-L is implemented by Intel as a frontier and highly optimized descendant of zlib, and in fact QPL’s deflate implementation is derived from ISA-L. What ISA-L does not have, however, is a hardware path that exploits Intel’s on-chip accelerators. QPL’s hardware path significantly outperformed every other library in the experiment, with a roughly 3× speedup over the second-best library in each category. The compression ratio, however, is slightly worse than even the other zlib-derived libraries, owing to the history buffer mismatch. Nevertheless, it’s clearly within the range of the zlib-derived libraries that are actively used in production code, and the speed-up is significant enough to warrant adoption.

**Table 1:** Summarized results of deflate compression algorithm benchmark.

Library	Compression time	Decompression time	Compression ratio
**Libdeflate**	4387	1318	0.424
**zlib**	9313	3244	0.448
**zlib-ng**	4108	2174	0.648
**ISA-L**	2354	1540	0.538
**QPL (SW path)**	2622	1750	0.685
**QPL (HW path)**	886	394	0.615

All timing values are reported to the closest second.

Moving on from benchmarking just the compression algorithm, we then tested QPL-HTSLib’s entire compression and decompression paths on simulated sequencing data generated from the GRCh38 reference genome using art_modern (Huang *et al.* 2012) at various read counts. A segment was taken from the beginning of each chromosome in this genome to create a smaller representative 100 MB dataset. Sample reads from this GRCh38 segment were then simulated using art_modern at fixed coverage levels (Huang *et al.* 2012). Each simulated dataset was compressed using both the standard Samtools build (linked against unmodified HTSLib) and custom builds where Samtools was linked against QPL-HTSLib in various test configurations. One of the files produced by QPL-HTSLib was then decompressed using each implementation as well. Various metrics were gathered throughout each test, including compression and decompression time and memory usage, and the original and compressed dataset file sizes. Each decompressed file was also validated to be identical to the original to confirm interoperability, which consistently matched across all trials.

**Figure 1:**
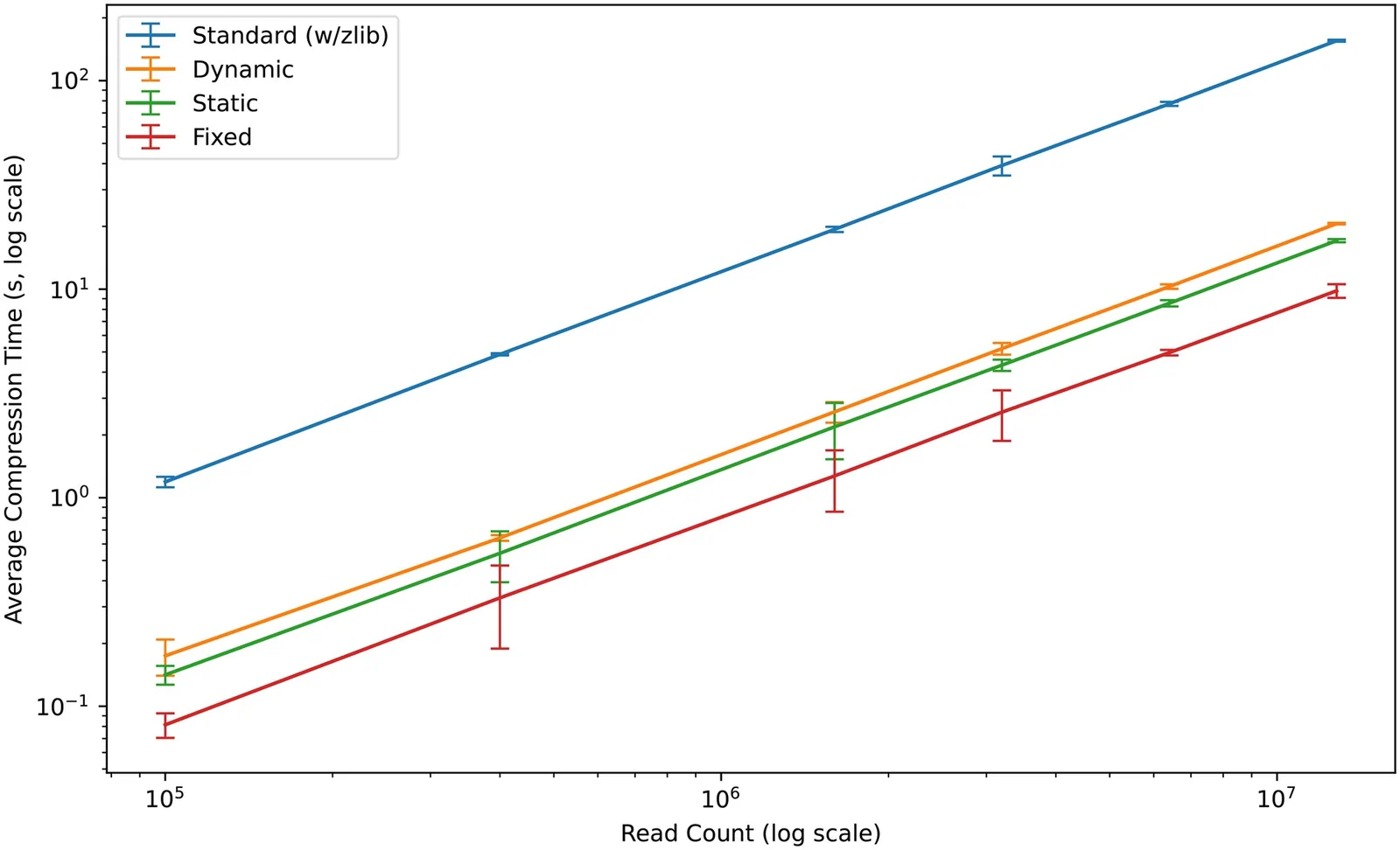
Huffman Building Mode Benchmark. Compression time for datasets simulated from a sample of GRCh38 Chromosome 1 using art_modern with *n *= 100K, 400K, 1.6M, 6.4M, and 25.6M mapped reads, comparing the standard HTSlib build (using zlib) against QPL-HTSLib configurations using dynamic, static, and fixed Huffman table construction. Both axes are logarithmically scaled.

QPL offers multiple configuration options for its compression mode. In order to evaluate the impact of these modes, we evaluated QPL-HTSLib across 50 simulated datasets: 10 each at 5 group levels offset by multiples of 4. Results are summarized in Table 2. The standard build of Samtools using zlib was compared to one utilizing QPL-HTSLib compiled with Dynamic Huffman mode (where the Huffman tree is dynamically determined per-block), one utilizing QPL-HTSLib compiled with a precalculated static huffman tree, and one compiled using a built-in but unoptimized fixed huffman tree. All 3 modes showed a significant speedup over the standard build, with fixed mode offering the highest speedup of 15× due to not requiring the calculation or loading of a tree. Static Huffman mode offered an 8.2× speedup, while Dynamic Huffman mode had a 7.4× speedup. However, the non-dynamic modes came at significant compression ratio cost due to their unoptimized encoding trees, and while Dynamic Huffman mode’s compression ratio was within 15% of the standard build, the Static and Fixed modes were 73% and 92% worse (Table 2). Thus, Dynamic Huffman mode offers the best overall results, and was chosen for QPL-HTSLib’s mode of operation. The 3 versions did not have significantly different decompression times or memory usage, so for its optimal mode, QPL-HTSLib offered a 7.4× speedup on compression and a 1.29× speedup on decompression. This performance gain came with a slight increase in memory usage and slightly lower compression ratio. As shown in Fig. 1, the time growth of all four methods was approximately equal and linear with respect to read count, so QPL-HTSLib remains a viable tool as file size scales. Differences in the underlying data led to larger standard deviations in the group-wise absolute timing analysis than in the relative comparison between configurations, highlighting that QPL-HTSLib offers comparable performance gains regardless of standard HTSLib’s efficiency on the data.

**Table 2:** Summarized results of huffman building mode benchmark.

HTSLib build	Compression time	Decompression time	Memory usage	Compression ratio
**Standard Build w/zlib**	**–**	**–**	6976±132	3.479±0.008
**Dynamic Compression**	0.135±0.007	0.778±0.06	9632±170	3.156±0.007
**Static Compression**	0.122±0.007	0.811±0.122	10767±189	1.674±0.004
**Fixed Compression**	0.066±0.006	0.797±0.101	10788±163	1.222±0.001

All timing values are reported relative to the zlib standard build. A single compression ratio and memory usage statistic is presented for each scenario because they were constant across all randomly generated datasets for each scenario.

**Figure 2:**
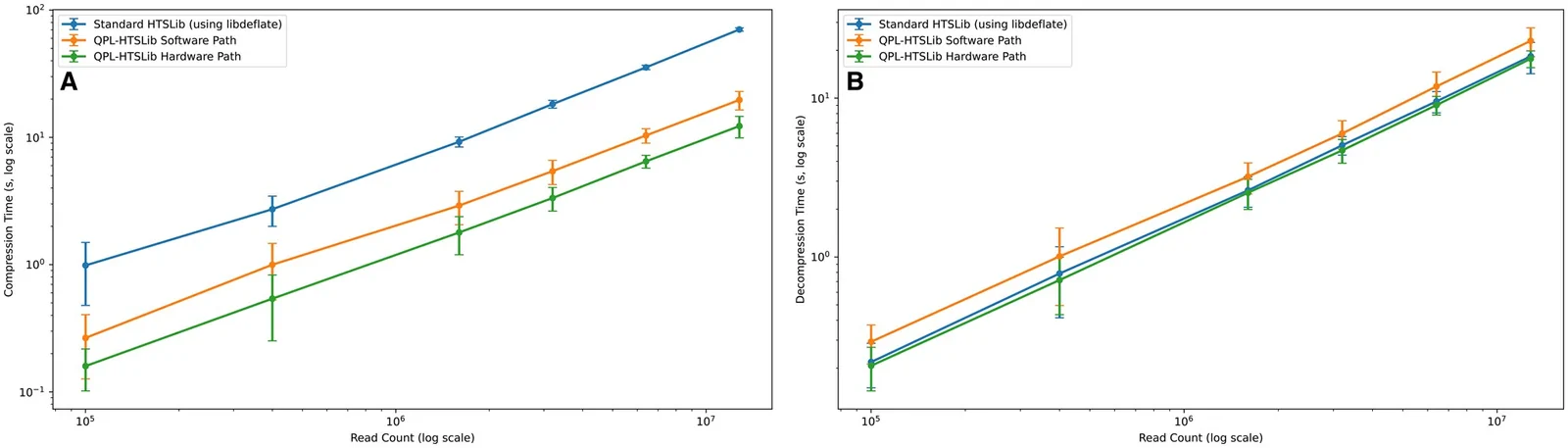
Hardware-Libdeflate Benchmark. (A) Compression time and (B) decompression time for art_modern simulated GRCh38 datasets with *n *= 100K, 400K, 1.6M, 3.2M, 6.4M, and 12.8M mapped reads, comparing the standard HTSlib build (using Libdeflate), QPL-HTSLib static software mode, and QPL-HTSLib static hardware mode. Both axes are logarithmically scaled. Error bands span ±3 standard deviations about the mean of [N] replicates.

In order to evaluate the performance of QPL-HTSLib using QPL’s software path versus its hardware path, we tested QPL-HTSLib on 300 more simulated datasets: 50 each at 6 read counts ranging from 100K to 12.8M. A different machine was used for this test as well, to evaluate the standard build using Libdeflate instead of zlib. Results are summarized in Table 3. On average, QPL-HTSLib’s software path was 3.376× faster than HTSLib using Libdeflate, which is consistent with Libdeflate providing HTSLib with a 2× speedup versus zlib as demonstrated in prior work (Siebelt 2024). QPL-HTSLib’s hardware path was a further 1.685× faster than the software path, for a total 5.580× faster performance than the standard build using libdeflate and an estimated 12.55× speedup compared to the standard build using zlib. For decompression, HTSLib using Libdeflate performed substantially better than the zlib version and was faster than QPL-HTSLib’s software path, though the hardware path still narrowly outperformed the standard build. QPL-HTSLib’s hardware path also outperformed its software path in terms of compression ratio, although the standard build maintained the highest compression ratio. As Fig. 2 shows, performance of the three builds remained linear in time with respect to size of the data being compressed or decompressed.

**Table 3:** Summarized results of hardware-libdeflate benchmark.

HTSLib build	Compression time	Decompression time	Memory usage (kB)	Compression ratio
**Standard Build w/libdeflate**	**–**	**–**	8530±351	3.804±0.008
**QPL-HTSLib Software Path**	0.305±0.05	1.266±0.192	11800±254	2.448±0.004
**QPL-HTSLib Hardware Path**	0.184±0.029	0.954±0.136	12900±381	2.689±0.003

All values are reported as mean ± standard deviation.

All timing values are reported relative to the libdeflate standard build, and memory usage is reported in kB. A single compression ratio is presented for each scenario because each ratio was constant across all randomly generated datasets.

To ground these results in real data, we also tested QPL-HTSLib’s software path against standard HTSLib using zlib on the same NA12878 ONT sequence dataset as was used for the Deflate Compression Algorithm Benchmark test. Standard HTSLib took 33 801 s (9 h, 23 m) to compress this dataset, and 2807 s (46 minutes) to decompress, yielding a compressed file size of 310.6GB. QPL-HTSLib compressed NA12878 in 3192 s (53 m), and decompressed it in 1963 s (33 m), yielding a compressed file size of 323.2GB. Thus, QPL-HTSLib exhibited a 10.59× speedup on compression and a 1.43× speedup on decompression, but with a 4.06% larger compressed file. These results validate the trends seen in previous experiments, with a massive gain on compression speed, an additional significant gain on decompression speed, but a slightly larger compressed file. However, because decompression is sped up so significantly, this somewhat offsets the practical impact of the lower compression ratio: a user who downloads and decompresses a large compressed dataset will spend slightly more time downloading the file due to the slightly increased compressed file size, but they will also save significant time during the decompress step when they actually use the data. Further, a single download of a BAM file is typically followed by multiple iterations of processing (and thus decompression), further compounding the practical runtime savings. Literature validates this idea: as Kryukov *et al.* described, “We attribute [the] enduring popularity [of gzip in sequence compression] to gzip’s wide availability, robustness and speed (especially decompression speed). These qualities appear to be able to outweigh gzip’s less than stellar compression ratio” (Kryukov *et al.* 2019).

Together, these results highlight QPL-HTSLib’s effectiveness across multiple evaluation axes. It integrates seamlessly with existing workflows, preserving full interoperability with standard SAM/BAM/CRAM tooling. Performance benchmarks across both software and hardware configurations demonstrate significant speedups in compression and decompression time over both zlib and libdeflate-based HTSlib builds. These improvements come with minimal memory overhead and acceptable tradeoffs in compression ratio, confirming QPL-HTSLib as a high-performance drop-in replacement for Intel-based genomics pipelines.

## Supplementary Material

vbag250_Supplementary_Data
